# Combination of bone marrow mesenchymal stem cells and moxibustion restores cyclophosphamide-induced premature ovarian insufficiency by improving mitochondrial function and regulating mitophagy

**DOI:** 10.1186/s13287-024-03709-0

**Published:** 2024-04-08

**Authors:** Ge Lu, Hong-xiao Li, Zi-wei Song, Jia Luo, Yan-liang Fan, Yao-li Yin, Jie Shen, Mei-hong Shen

**Affiliations:** 1 College of Acupuncture Moxibustion and Tuina, Nanjing University of Chinese Medicine, Nanjing, China; 2 Key Laboratory of Acupuncture and Medicine Research of Ministry of Education, Nanjing University of Chinese Medicine, Nanjing, China

**Keywords:** Premature ovarian insufficiency, Bone marrow mesenchymal stem cells, Moxibustion, Mitochondrial function, Mitophagy

## Abstract

**Background:**

Premature ovarian insufficiency (POI) is a major cause of infertility. In this study, we aimed to investigate the effects of the combination of bone marrow mesenchymal stem cells (BMSCs) and moxibustion (BMSCs-MOX) on POI and evaluate the underlying mechanisms.

**Methods:**

A POI rat model was established by injecting different doses of cyclophosphamide (Cy). The modeling of POI and the effects of the treatments were assessed by evaluating estrous cycle, serum hormone levels, ovarian weight, ovarian index, and ovarian histopathological analysis. The effects of moxibustion on BMSCs migration were evaluated by tracking DiR-labeled BMSCs and analyzing the expression of chemokines stromal cell-derived factor 1 (*Sdf1*) and chemokine receptor type 4 (*Cxcr4*). Mitochondrial function and mitophagy were assessed by measuring the levels of reactive oxygen species (ROS), mitochondrial membrane potential (MMP), ATP, and the mitophagy markers (*Drp1*, *Pink1*, and Parkin). Furthermore, the mitophagy inhibitor Mdivi-1 and the mitophagy activator CCCP were used to confirm the role of mitophagy in Cy-induced ovarian injury and the underlying mechanism of combination therapy.

**Results:**

A suitable rat model of POI was established using Cy injection. Compared to moxibustion or BMSCs transplantation alone, BMSCs-MOX showed improved outcomes, such as reduced estrous cycle disorders, improved ovarian weight and index, normalized serum hormone levels, increased ovarian reserve, and reduced follicle atresia. Moxibustion enhanced *Sdf1* and *Cxcr4* expression, promoting BMSCs migration. BMSCs-MOX reduced ROS levels; upregulated MMP and ATP levels in ovarian granulosa cells (GCs); and downregulated *Drp1*, *Pink1*, and Parkin expression in ovarian tissues. Mdivi-1 significantly mitigated mitochondrial dysfunction in ovarian GCs and improved ovarian function. CCCP inhibited the ability of BMSCs-MOX treatment to regulate mitophagy and ameliorate Cy-induced ovarian injury.

**Conclusions:**

Moxibustion enhanced the migration and homing of BMSCs following transplantation and improves their ability to repair ovarian damage. The combination of BMSCs and moxibustion effectively reduced the excessive activation of mitophagy, which helped prevent mitochondrial damage, ultimately improving ovarian function. These findings provide a novel approach for the treatment of pathological ovarian aging and offer new insights into enhancing the efficacy of stem cell therapy for POI patients.

**Graphical abstract:**

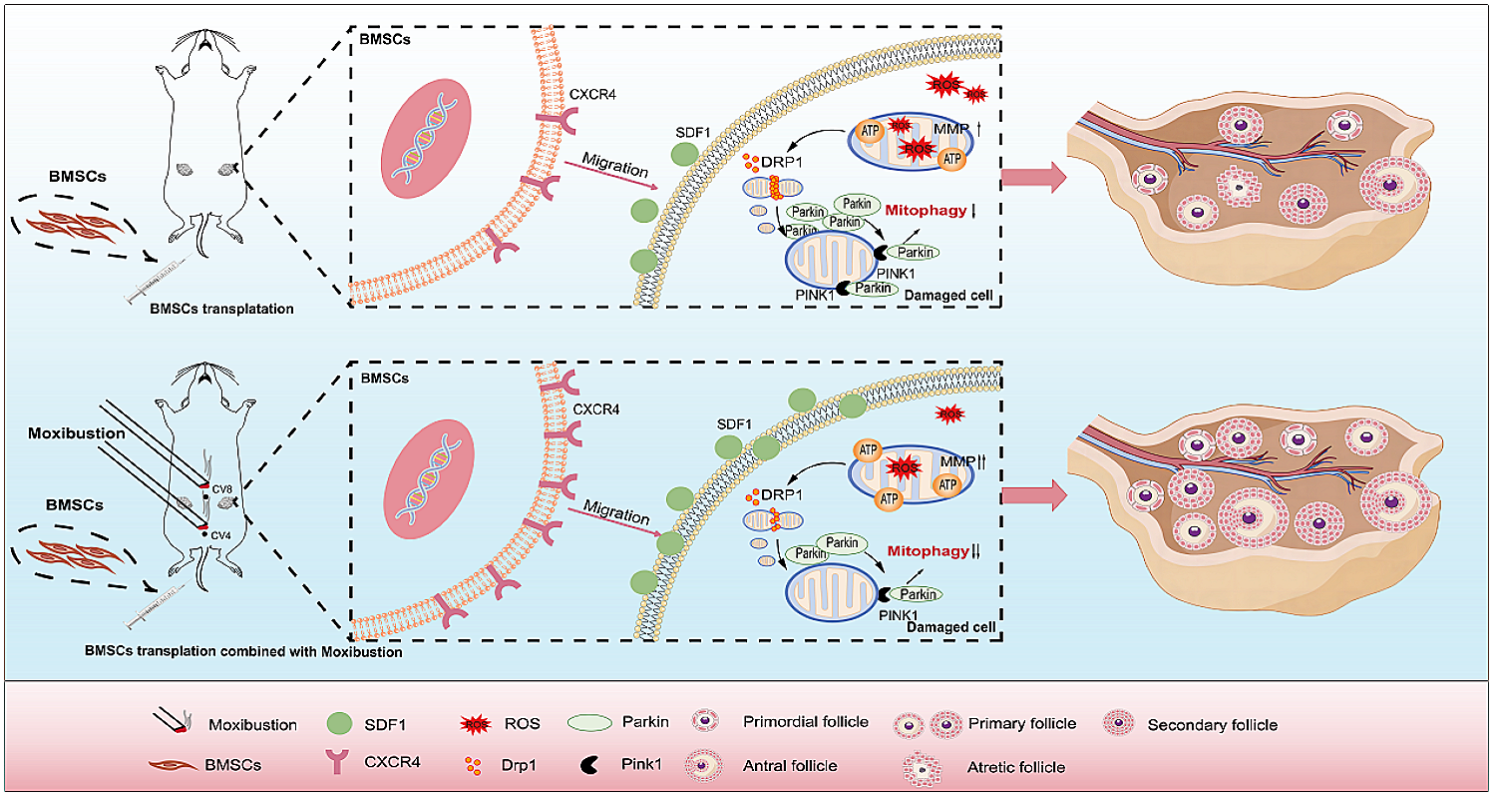

**Supplementary Information:**

The online version contains supplementary material available at 10.1186/s13287-024-03709-0.

## Introduction

The ovary, as a crucial component of the female reproductive system, is one of the earliest organs to display aging-associated dysfunction. Ovarian aging primarily manifests as a decreased number and quality of oocytes in the ovarian cortex [1]. Physiological aging is an inevitable process; due to various genetic, medical, and environmental factors, the ovary may experience pathological aging. Pathological ovarian aging encompasses reduced ovarian reserve, premature ovarian insufficiency (POI), and poor ovarian response [2]. POI, which has become the primary focus of research on ovarian aging, refers to the development of ovarian decline in women before the age of 40 years [3, 4]. Hormone replacement therapy is the main treatment for ovarian aging, especially for POI. It involves the use of hormones to alleviate low estrogen symptoms [5]. However, hormone replacement therapy is associated with numerous adverse reactions and potential risks and does not improve ovarian function and pregnancy rates [6–8]. Even with assisted reproductive technology, clinical pregnancy outcomes in patients with ovarian aging are not favorable [9]. Therefore, finding effective fertility protection measures to delay pathological ovarian aging and treat POI remains challenging.

Mesenchymal stem cells (MSCs) have emerged as potential therapeutic candidates for ovarian aging [10, 11]. A recent study provided clinical evidence that substantiated the effects of MSCs in improving ovarian response, pregnancy rate, and live birth rate [12]. Bone marrow mesenchymal stem cells (BMSCs) are advantageous owing to their easy accessibility and low immunogenicity [13, 14]. BMSCs can restore ovarian endocrine function and improve ovulation by migrating to damaged tissues and regulating various signaling pathways [13, 15], thereby enhancing fertility. However, the clinical applications of stem cell therapy are hindered by challenges, such as an unfavorable microenvironment at the damaged site, insufficient transplanted cells, and a low survival rate (less than 20%) [16], limiting the widespread use of stem cell therapy. Combining electroacupuncture (EA) with MSCs transplantation has been shown to improve the survival and therapeutic effects of MSCs [17]. Similar to EA, moxibustion is another traditional Chinese medicine therapy that stimulates acupoints and meridians by applying heat from moxibustion fires, near-infrared stimulation, and special substances found in moxa smoke. Previous studies have demonstrated that moxibustion can enhance fertility in patients with diminished ovarian reserve function by acting on the reproductive system through various channels and targets [18–20]. However, it remains unclear whether moxibustion can improve the survival, migration, and therapeutic effects of MSCs. Therefore, it is worth investigating whether combining BMSCs with moxibustion can result in better efficacy in improving ovarian function.

Previous studies have established a strong correlation between ovarian aging and mitochondrial dysfunction [21, 22]. Mitochondria are particularly vulnerable to peroxidative damage caused by excessive reactive oxygen species (ROS), being the primary source of cellular ROS production [23]. Excessive accumulation of ROS can disrupt mitochondrial membrane permeability, resulting in structural abnormalities and impaired functionality. Mitophagy, which involves the selective wrapping and degradation of damaged or dysfunctional mitochondria within cells, plays a crucial role in maintaining the stability of the mitochondrial network and intracellular environment [24]. However, inactive or excessive mitophagy activation is detrimental [25, 26]. Thus, targeting mitophagy will bring great benefit to mitochondrial function [27]. While whether the therapeutic effect of BMSCs and moxibustion on POI is associated with mitophagy regulation is currently unclear. Therefore, in this study, we established an appropriate animal model of POI and aimed to investigate the effects of moxibustion on BMSCs and the role of moxibustion combined with BMSCs in the treatment of chemotherapeutic drug-induced pathologic ovarian aging and the potential mechanisms involved.

## Materials and methods

### Animals and treatment

In this study, we used female Sprague–Dawley rats (aged 8 weeks; weight, 190–210 g) from Zhejiang Vital River Laboratory Animal Technology Co., Ltd. (Zhejiang, China). All animals were maintained at 23 ± 2 °C, 55 ± 5% relative humidity, and a fixed 12-h light/dark cycle in the Laboratory Animal Center of Nanjing University of Chinese Medicine.

In this study, we assessed the effects of different doses of cyclophosphamide (Cy) (Sigma-Aldrich，C0768) on ovarian aging to establish a rat model of POI. Forty-eight rats with regular estrous cycles were randomly divided into four groups:

1. Group 1 (CON): treated with 0.9% saline, *n* = 12.
2. Group 2 (POI-1): treated with 25 mg/kg of Cy for Day 1 and 8 mg/kg of Cy for the next 14 consecutive days, *n* = 12.
3. Group 3 (POI-2): treated with 50 mg/kg of Cy for Day 1 and 8 mg/kg of Cy for the next 14 consecutive days, *n* = 12.
4. Group 4 (POI-3): treated with 100 mg/kg of Cy for Day 1 and 8 mg/kg of Cy for the next 14 consecutive days, *n* = 12.

To confirm the successful establishment of the POI rat model, we assessed various factors, including the estrous cycle, serum hormone levels, ovarian weight and index, ovarian morphological changes, and follicle counting, in each group.

Additionally, we evaluated the combinatorial effect of BMSCs and moxibustion on restoring ovarian function, mitochondrial function, and mitophagy. Rats with regular estrous cycles were randomly divided into five groups: CON, POI, MOX, BMSCs, and BMSCs-MOX (*n* = 20). The CON group received a similar dose of 0.9% saline, and other groups received intraperitoneal injections of Cy to induce POI. After the modeling process, rats in the BMSCs and BMSCs-MOX groups received tail vein injections of BMSCs on Day 1, Day 3, and Day 7, and rats in other groups received similar doses of phosphate-buffered saline (PBS). Rats in the MOX and BMSCs-MOX groups underwent anesthesia, grasping, and moxibustion treatment for 15 days, and those in the remaining groups were subjected to the same anesthesia and grasping procedures without moxibustion.

To investigate the impact of mitophagy on Cy-induced ovarian aging and its role in BMSCs-MOX treatment, we divided the rats into five groups: CON, POI, Mdivi-1, BMSCs-MOX, and BMSCs-MOX-CCCP (*n* = 20). In the Mdivi-1 group, rats were administered a daily intraperitoneal injection of mitophagy inhibitor (Mdivi-1) (1.2 mg/kg qd), and other interventions were same as those in the POI group. In addition to the treatment received by the rats in the BMSCs-MOX group, the rats in the BMSCs-MOX-CCCP group were also injected intraperitoneally with mitophagy activator (CCCP) (2 mg/kg qod).

All animal experiments were approved by the Animal Experiment Ethics Committee of the Nanjing University of Chinese Medicine (No. 202106A03, No. 202204A023).

### BMSCs preparation, culture, and identification

BMSCs were isolated from the bone marrow of 3-week-old male rats using the whole-bone marrow adherence method. Briefly, the rats were sacrificed to collect the femurs and tibias. Bone marrow cells were collected by rinsing the bone marrow chambers with L-DMEM containing 10% FBS (Oricell, FBSSR-01021) and 1% penicillin-gentamicin (Basal Media, S110 KJ). The cell pellets were resuspended and seeded in T75 flasks after centrifugation at 1,500 rpm for 5 min. Non-adherent hematopoietic cells were removed after 24 h, and the culture medium was changed every 2 days. The cells were passaged when they reached 80–90% confluence.

Third-generation BMSCs were collected for flow cytometry, and osteogenic and adipogenic differentiation assays to identify cell surface markers (CD29-FITC, CD90-FITC, CD11b-PE, CD45-PE) (Invitrogen, 11-0291-80, 11-0900-8, 12-0110-80, 12-0461-80) and the pluripotency of MSCs. All subsequent experiments were performed using BMSCs from passages three to five (P3 to P5).

### DiR-labeled BMSCs tracking

To track and localize the BMSCs transplanted into rat tissues, we labeled them with DiR iodide (Yeasen, 40757ES25). Briefly, we selected well-growing BMSCs from the P3 to P5 generations and prepared a cell suspension (1 × 10^6^ cells/mL) after digestion and centrifugation. The cells were then incubated with a 2 µg/mL DiR staining solution at 37℃ for 20 min. After incubation, the cells were rinsed, resuspended in PBS, and transplanted into rats via the tail vein.

On days 1, 3, and 7 after BMSCs transplantation, two randomly selected rats from the BMSCs and BMSCs-MOX groups were observed for BMSCs migration using an in vivo fluorescence imaging system (PerkinElmer, IVIS Spectrum). Frozen ovarian sections were used to observe the colonization and distribution of BMSCs in the ovarian tissues.

### Moxibustion treatment

All rats were anesthetized with sodium pentobarbital. The MOX, BMSCs-MOX, and BMSCs-MOX-CCCP groups were treated with moxibustion. The moxibustion treatment methods have been described in previous studies [28]. Two groups of acupoints were selected: one included bilateral Shenshu (BL23), and the other included Guanyuan (CV4) and Shenque (CV8). One acupoint group was selected per day. During moxibustion treatment, a moxibustion stick (diameter, 5.3 mm; length, 85 mm) was aimed at the acupoints positioned 1 cm above them. Moxibustion treatment was performed once a day for 10 min each, with continuous intervention for 15 days. The rats in the remaining groups were subjected to the same anesthesia and grasping procedures without moxibustion.

### Estrous cycle

Vaginal smears were used to monitor the estrous cycle. Based on previous studies [18, 19, 28], the estrous cycle was categorized into four stages: pre-estrus (P), estrus (E), metestrus (M), and diestrus (D). Estrous cycle disorder is defined as a significantly prolonged cycle (≥ 6 days) or no change in a particular stage for ≥ 3 days.

### Serum hormone levels

The serum levels of estradiol (E_2_), follicle-stimulating hormone (FSH), luteinizing hormone (LH), and anti-Müllerian hormone (AMH) were measured using commercially available ELISA kits (Elabscience, E-OSEL-R0001, E-EL-R0391c, E-EL-R0026c, and E-EL-R3022) according to the manufacturer’s instructions.

### Ovarian morphology and follicle counting

Ovaries were embedded in paraffin blocks, fixed, and dehydrated. A series of 6-µm-thick sections were then prepared, and 1 out of 10 slides was stained with hematoxylin and eosin (H&E) to evaluate ovarian morphology and the number of follicles at different stages, as previously described [18]. All procedures were performed under a light microscope.

### Immunohistochemistry (IHC)

During IHC, the ovarian sections underwent the following steps: dewaxing to water, blocking endogenous peroxides, antigen repair, closure, primary and secondary antibody incubation, DAB color development, hematoxylin re-staining, dehydration, transparency, and sealing. The sections were observed under a light microscope. Five fields of view (400×) were randomly selected for photography on each tissue section. The mean optical density (MOD) values of the target proteins in the ovarian tissues were measured using ImageJ software. The primary antibodies used in this study are listed in Additional file 1: Table S1.

### Western blotting

Protein samples extracted from the ovaries were separated using SDS-PAGE (25 µg/well) and transferred to PVDF membranes. Following blocking in 5% skim milk, the membranes were incubated with primary antibodies overnight at 4℃ and secondary antibodies at room temperature the next day. Immunoreactive bands were detected using Super ECL detection reagent (Fude, FD8020), and images were captured using a Chemiluminescence imaging system (Bio-Rad, Chemi Doc XRS+). The primary antibodies used in this study are listed in Additional file 1: Table S1.

### Quantitative reverse-transcription polymerase chain reaction (PCR) (qRT-PCR)

Total RNA was extracted from the ovarian tissues, and cDNA was synthesized by the Hifair® II 1st Strand cDNA Synthesis Kit (Yeasen, 11141ES60) for qRT-PCR. The relative quantifications were performed using Hieff® qPCR SYBR Green Master Mix (Low Rox Plus) (Yeasen, 11202ES08) in a quantitative PCR instrument (Agilent, Stratagene Mx3000P). *Gapdh* was detected as the internal reference control gene, and the relative expression levels of mRNAs were determined by the 2 ^− ΔΔCT^ method. The primers used in this study are listed in Additional file 2: Table S2.

### Collection of ovarian granulosa cells (GCs) in the ovary

Ovarian GCs were collected following established methods from a previous study [28]. Briefly, the bilateral rat ovaries were isolated and placed in pre-cooled PBS. Ovarian follicles were punctured using small needles under a stereomicroscope (Jiangnan, JSZ6) to release the GCs. The collected suspension was centrifuged at 1,000 rpm for 5 min. The cells were resuspended in PBS, and cell density was adjusted to 1 × 10^6^ cells/mL for subsequent experiments.

### Measurement of ROS levels

ROS levels in GCs were measured using the ROS Assay Kit (Jiancheng, E004-1-1). GCs were incubated with 10 mM dichlorofluorescein diacetate at 37℃ for 30 min. ROS levels were measured at 488 nm using a multicolor analytical flow cytometer (Beckman, Gallios). Data were analyzed using FlowJo software v10.6.2.

### Measurement of mitochondrial membrane potential (MMP)

MMP in GCs was assessed using an enhanced MMP assay kit with the JC-1 dye (Beyotime, C2003s). Following the manufacturer’s instructions, GCs were incubated with JC-1 working solution at 37 °C for 20 min. After rinsing with the JC-1 buffer solution, GCs were detected using a multicolor analytical flow cytometer (Beckman, Gallios). Data were analyzed using FlowJo software v10.6.2.

### ATP detection

ATP levels in GCs were measured using an ATP Assay Kit (Beyotime, S0026). GCs were lysed with an ATP lysis buffer and mixed with an ATP detection working solution. The relative fluorescence intensities were measured using a multifunctional enzyme labeler (PerkinElmer, EnVision), and the ATP concentration of the samples was calculated according to the corresponding standard curve.

### Statistical analysis

The results were analyzed using SPSS 26.0. Counting data are presented as numbers and percentages, and measurement data are expressed as mean ± standard deviation. First, a normal distribution test was performed. For data that followed a normal distribution, one-way ANOVA was used, and nonparametric tests were performed for data that did not conform to a normal distribution. The LSD method was used for group comparisons of Chi-square conforming data, and Tamhane’s T2 method was used for Chi-square non-conforming data. Fisher’s exact test was used to analyze the rate of estrous cycle disorders. Two-sided tests were performed, and differences were considered statistically significant at *P* < 0.05.

## Results

### Isolation and identification of BMSCs

**Fig. 1:**
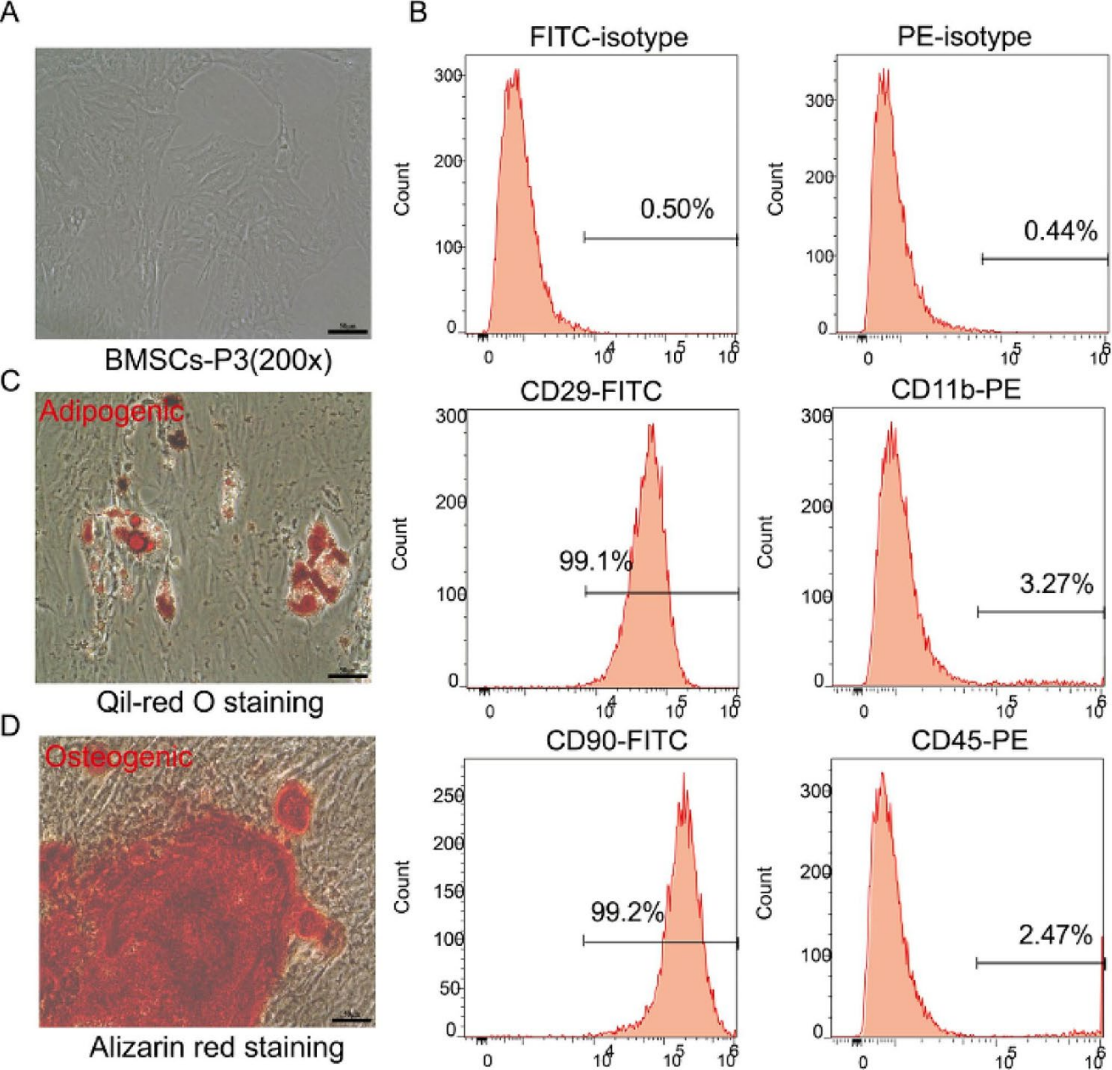
Isolation and identification of BMSCs. **A** The isolated BMSCs exhibit typical spindle or shuttle-like morphology. Bar: 50 μm. **B** BMSCs show strong positive expression of CD29 and CD90 and negative expression of CD11b and CD45. **C** Induction of BMSCs into adipocytes. Bar: 50 μm. **D** Induction of BMSCs into osteoblast. Bar: 50 μm

BMSCs were isolated from the bone marrow aspirates of 3-week-old male rats and cultured until the third passage. The BMSCs exhibited a typical spindle or shuttle-like morphology (Fig. 1A) and strong positive expression of CD29 and CD90 but a negative expression of CD11b and CD45 (Fig. 1B). Oil Red O and Alizarin Red staining confirmed the ability of the BMSCs to differentiate into adipocytes and osteocytes, indicating their multipotency (Fig. 1C–D). These findings warranted the use of BMSCs in subsequent experiments.

### Cy injection causes acute ovarian damage

**Fig. 2:**
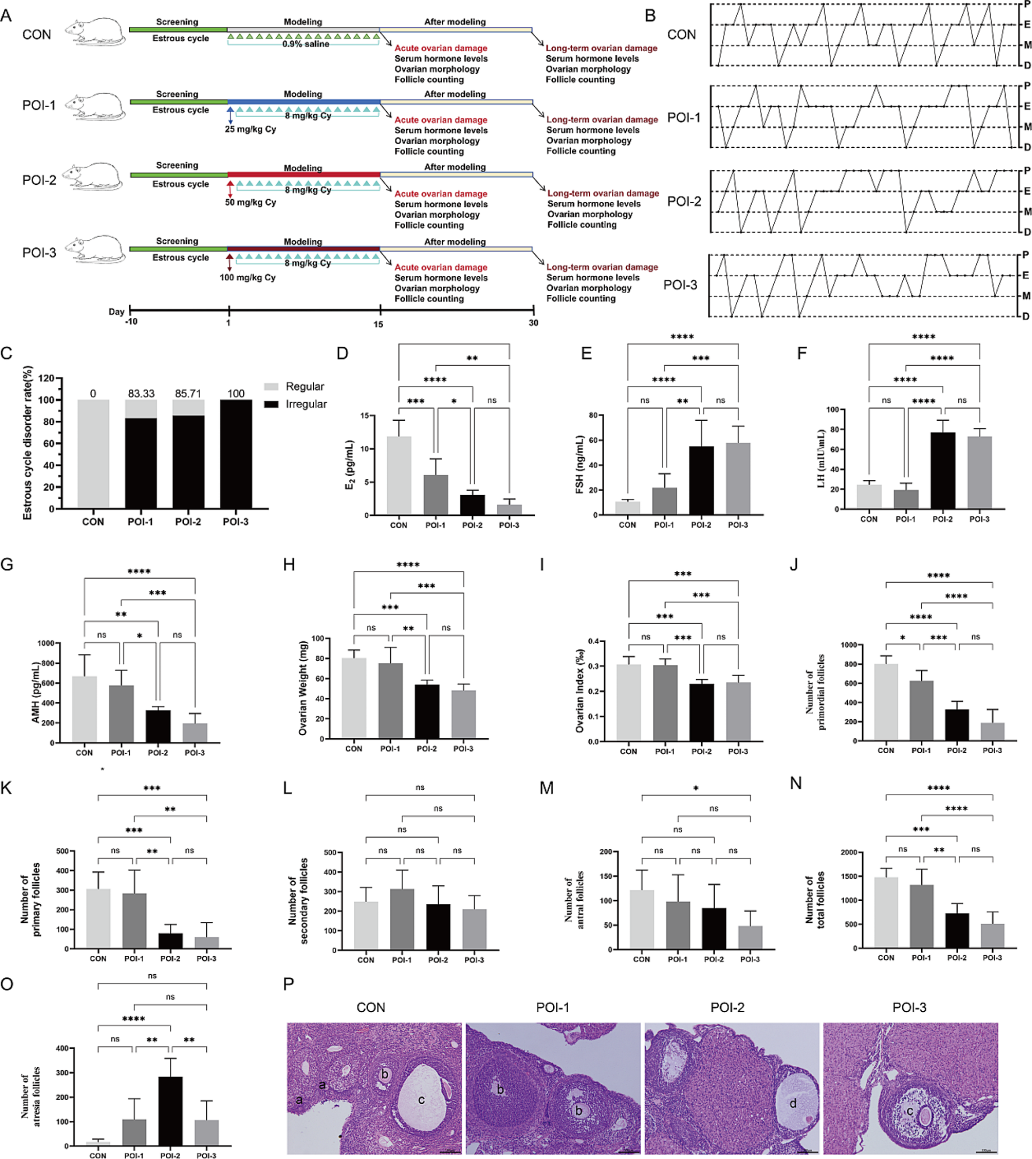
Cy injection causes acute ovarian damage. **A** The establishment process of the POI rat model. **B** Representative images of estrous cycles. **C** The rates of estrous cycle disorder. **D–G** Serum levels of E_2_ (D), FSH (E), LH (F), AMH (G) (*n* = 6). **H–I** Ovarian weights and indexes (*n* = 6). Ovarian index (‰) = ovarian weight (mg)/rat weight (g) × 1,000‰. **J–M** The numbers of primordial (J), primary (K), secondary (L), and antral follicles (M) (*n* = 6). N The numbers of total follicles (*n* = 6). **O** The numbers of atresia follicles (*n* = 6). **P** The structure of ovaries. **a** Representative primordial follicles, **b** Representative secondary follicles, **c** Representative antral follicles, and **d** Representative atresia follicles. Bar: 100 μm.*P < 0.05, ** *P* < 0.01, *** *P* < 0.001; **** *P* < 0.0001

Exposure to Cy usually leads to acute impaired endocrine function and ovarian damage [23]. The extent of ovarian damage depends primarily on the toxicity and dosage of Cy. We first evaluated the acute injury to the whole body and ovaries caused by different doses of Cy (Fig. 2). All doses of Cy resulted in a significant decline in the body weight of rats (Fig. S1A). Some rats in the POI-2 and POI-3 groups exhibited symptoms, such as depression, fur shedding, and mucous membrane hemorrhage (Fig. S1B). Survival rate analysis indicated that more than 50% of the rats in the POI-3 group were unable to tolerate the full round of Cy injection. In contrast, significantly higher survival rates were observed in the POI-1 and POI-2 groups (Fig. S1C).

Following the assessment of the estrous cycle disorder rate, an important measure of female reproductive function [29], none of the rats in the CON group exhibited disorder in their estrous cycles throughout the study. However, when various doses of Cy were administered intraperitoneally, 83.33% of the rats in the POI-1 group, 85.71% in the POI-2 group, and all rats in the POI-3 group developed irregular estrous cycles (Fig. 2B–C).

To assess the effect of Cy on hormone secretion, we measured the serum levels of E_2_, FSH, LH, and AMH using ELISA. Cy injection significantly decreased the serum E_2_ levels in POI-1 rats but did not notably affect serum FSH, LH, or AMH levels relative to the levels in the CON group. In the POI-2 and POI-3 groups, serum E_2_ and AMH levels were significantly lower than those in the CON group, whereas the serum levels of FSH and LH were significantly higher (Fig. 2D–G).

To directly evaluate the damage caused by Cy to the ovary, we assessed the ovarian weight and index and performed H&E staining of ovarian sections to observe structural changes in the ovary and calculate the number of follicles at all levels. As shown in Fig. 2H–P, the ovaries of rats in the CON group exhibited heavier and plumper morphology, with various stages of follicles in the ovarian cortex. Different doses of Cy resulted in smaller ovaries and decreased numbers of primordial (Fig. 2J) and total follicles (Fig. 2N). There was a significant decrease in the number of primary follicles (Fig. 2J) and an increase in the number of atretic follicles in the POI-2 group (Fig. 2O). These findings indicated that different doses of Cy impaired endocrine function and caused acute ovarian damage.

### Medium and high doses of Cy cause long-term premature ovarian aging

**Fig. 3:**
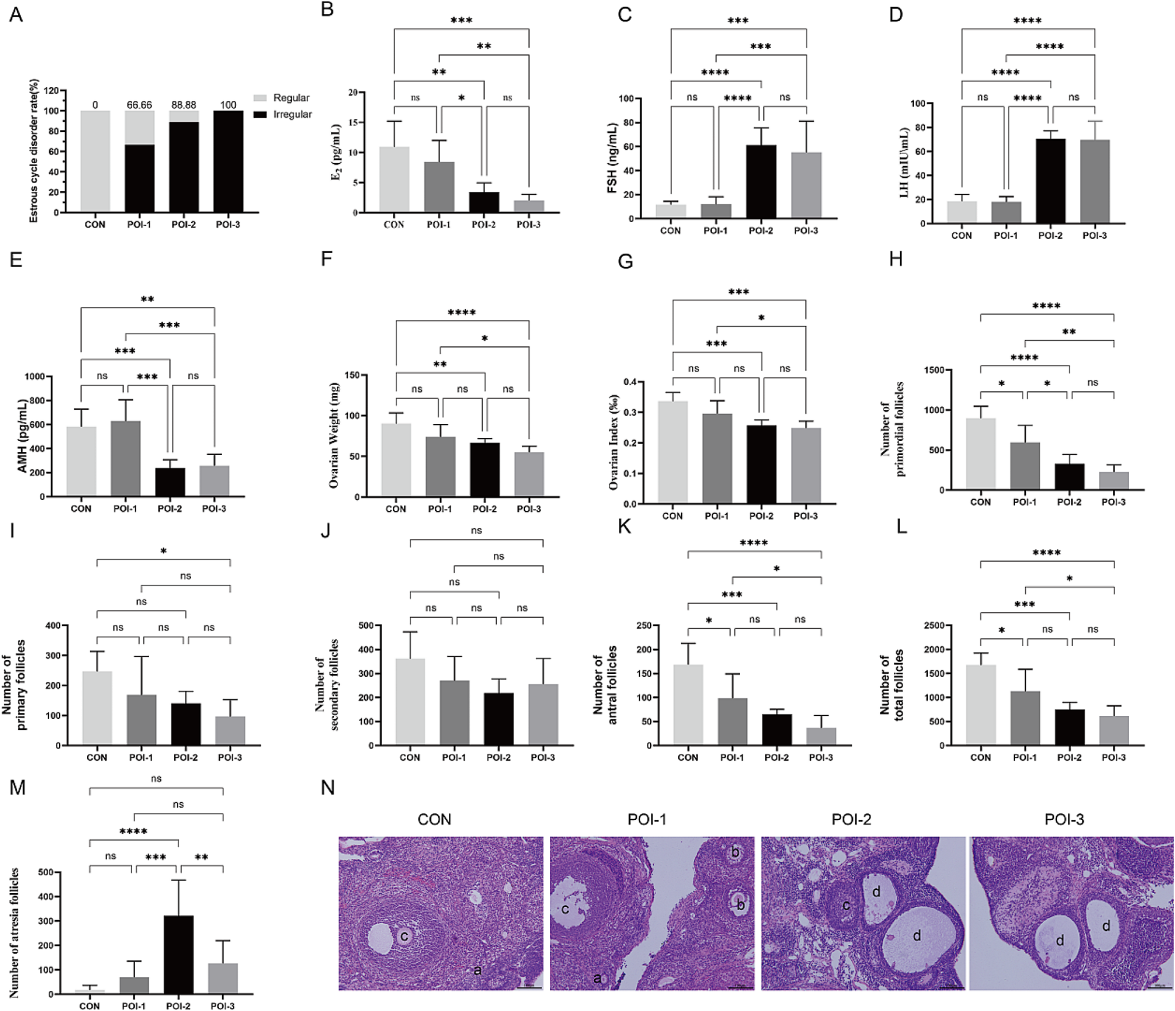
Medium and high doses of Cy cause long-term premature ovarian aging. **A** The rate of disorder estrous cycle disorders. **B–E** Serum levels of E_2_ (B), FSH (C), LH (D), and AMH (E) (*n* = 6). **F–G** Ovarian weights and indexes (*n* = 6). **H–K** The numbers of follicles in different levels (*n* = 6). **L** The numbers of total follicles (*n* = 6). **M** The numbers of atresia follicles (*n* = 6). **N** The structure of ovaries. **a** Representative primordial follicles, **b** Representative secondary follicles, **c** Representative antral follicles, and **d** Representative atresia follicles. Bar: 100 μm. * *P* < 0.05, ** *P* < 0.01, *** *P* < 0.001; **** *P* < 0.0001

To assess whether Cy contributed to long-term premature ovarian aging, we examined ovarian and endocrine function in rats 15 days after Cy injection. A decrease in the rate of estrous cycle disorders was observed in the POI-1 group, some of which recovered with regular estrous cycles (Fig. 3A). However, no significant improvements were observed in the POI-2 and POI-3 groups (Fig. 3A). No significant differences in the serum hormone levels were observed between the POI-1 and CON groups (Fig. 3B–E). In contrast, rats in the POI-2 and POI-3 groups exhibited remarkably decreased serum E_2_ and AMH levels and significantly elevated serum FSH levels compared with those in the CON group (Fig. 3B–E). Ovarian histomorphology evaluations further confirmed the lasting damage caused by medium and high doses of Cy, as indicated by a decrease in ovarian weight and the number of primordial, primary, and total follicles (Fig. 3F–N). The POI-2 group showed a significant increase in the number of atretic follicles compared with the CON group. These observations support that medium and high doses of Cy cause long-term ovarian premature aging. Considering animal survival, we observed that a medium dose of Cy was practical for constructing a POI rat model with a high success rate, low mortality, and good reproducibility.

### Moxibustion promotes BMSCs migration

**Fig. 4:**
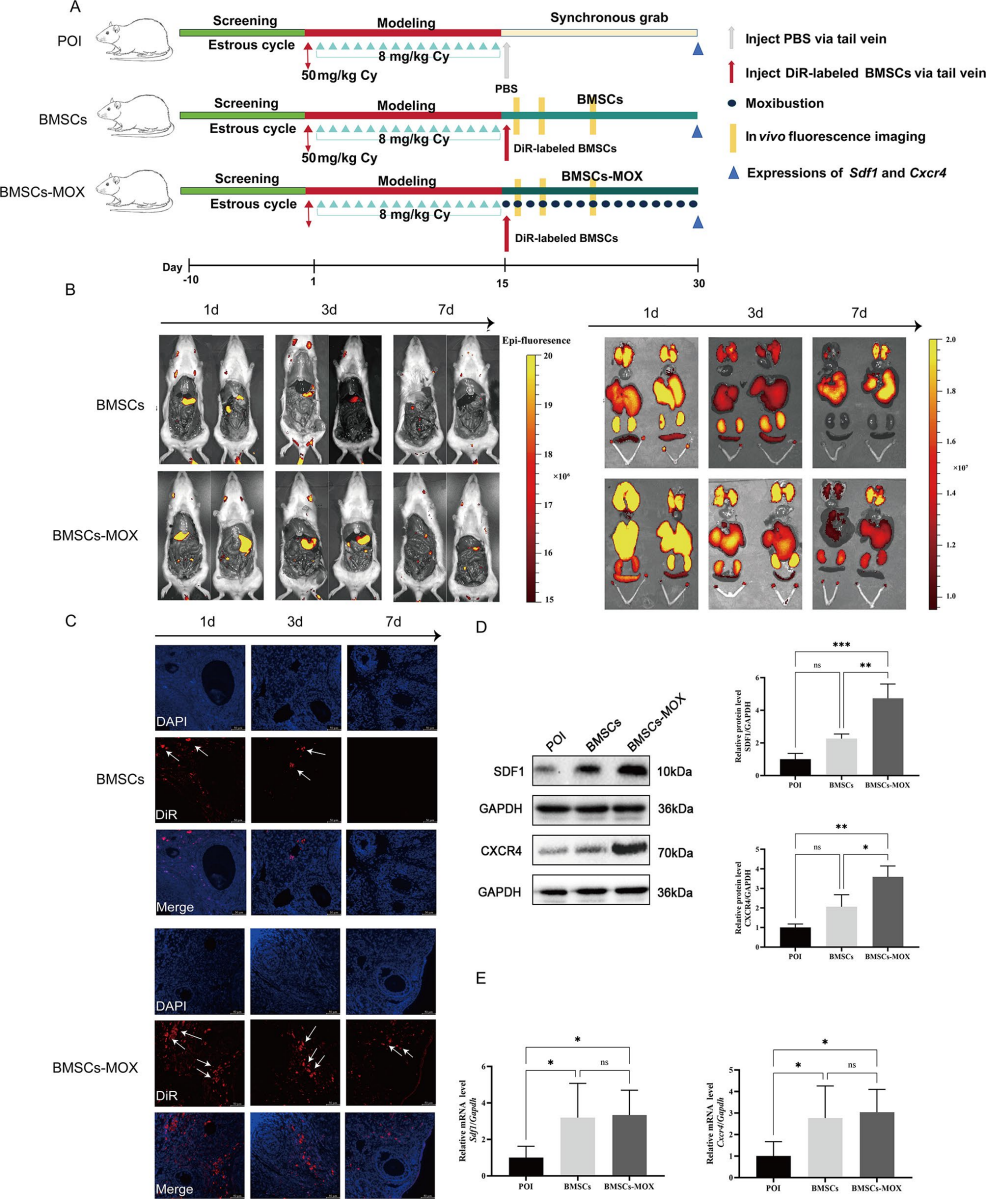
Moxibustion promotes BMSCs migration. **A** The process of BMSCs tracking. **B–C** DiR-labeled BMSCs tracking (in-vivo fluorescence imaging and ovarian frozen sections). White arrow represents DiR-labeled BMSCs. **D** SDF1 and CXCR4 protein levels (*n* = 3). Full-length blots are presented in Additional file 4: Fig. S2. E *Sdf1* and *Cxcr4* mRNA expression (*n* = 6). * *P* < 0.05, ** *P* < 0.01, *** *P* < 0.001

To investigate the migration of transplanted BMSCs into damaged ovarian tissue, we labeled BMSCs with DiR and tracked the fluorescent cells in vivo (Fig. 4A–B). On the first day after transplantation, significant fluorescence was observed in the lung, liver, kidney, and ovary tissues of the BMSCs and BMSCs-MOX groups. By the third day, the fluorescence intensity in the ovarian tissue of the BMSCs-MOX group was significantly higher than that of the BMSCs group. On the seventh day, the fluorescence in the ovarian tissue of the BMSCs group weakened considerably, and the BMSCs-MOX group still exhibited significant fluorescence. These observations indicated that moxibustion stimulation promoted the accumulation of BMSCs in damaged tissues.

Stromal cell-derived factor 1 (*Sdf1*) and C-X-C chemokine receptor type 4 (*Cxcr4*) are crucial regulators of MSCs migration [30]. To determine the role of MOX in promoting the accumulation of BMSCs in the ovaries, we evaluated *Sdf1* and *Cxcr4* expression. As shown in Fig. 4D, no significant differences were observed in SDF1 and CXCR4 protein levels in the ovaries between the BMSCs and POI groups; in contrast, these levels were significantly increased in the BMSCs-MOX group. Regarding mRNA expression, BMSCs and BMSCs-MOX interventions increased *Sdf1* and *Cxcr4* mRNA levels in the ovaries (Fig. 4E). Therefore, the increased accumulation of BMSCs in the ovarian tissue might be attributed to the enhanced expression of Sdf1 and Cxcr4 after moxibustion stimulation.

### BMSCs-MOX restores hormone secretion and reduces the pathological changes in Cy-induced POI

**Fig. 5:**
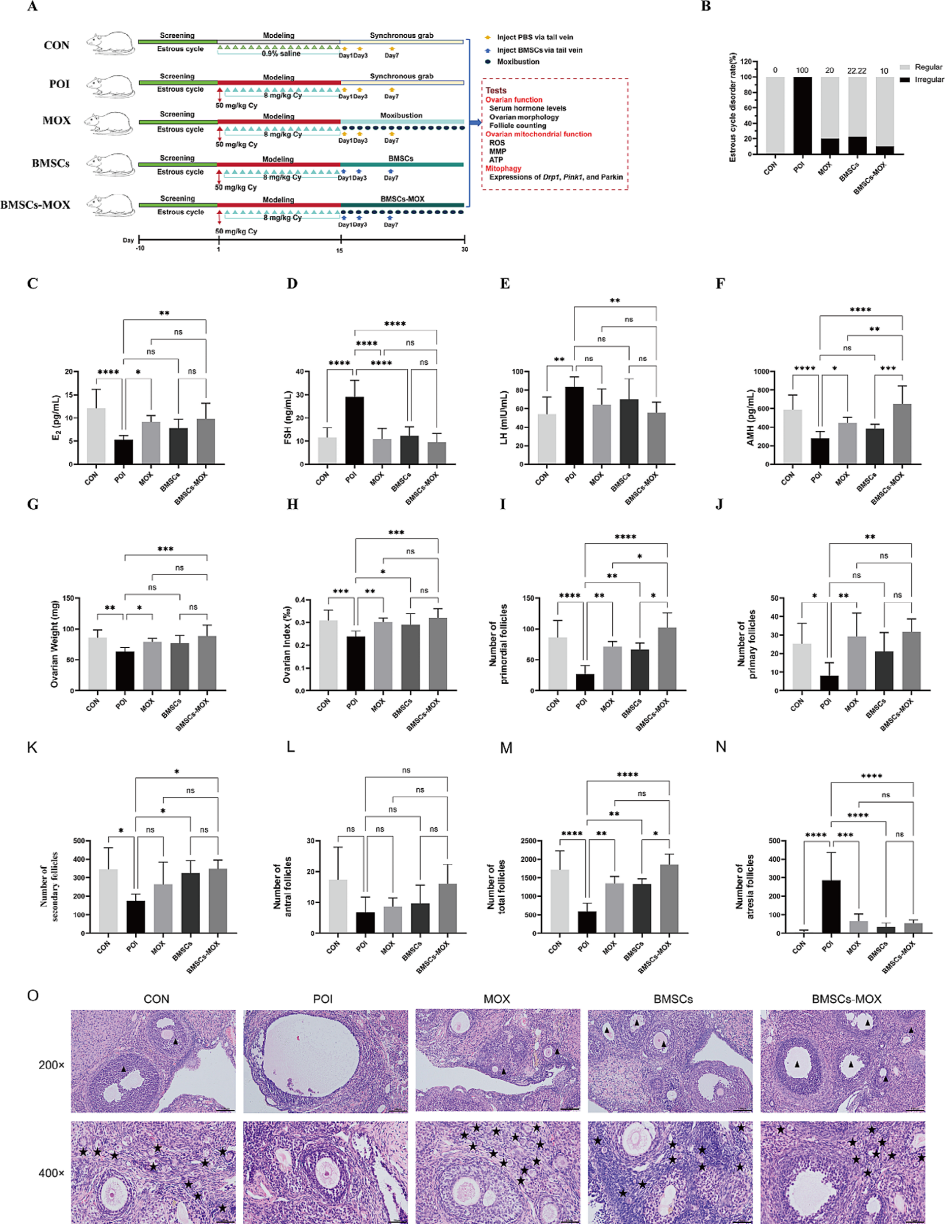
BMSCs-MOX restores hormone secretion and reduces the pathological changes in Cy-induced premature ovarian aging. **A** The process of BMSCs transplantation and moxibustion treatment. **B** The rate of estrous cycle disorder (*n* = 10, except for POI group [*n* = 8], BMSCs [*n* = 9]). **C**–**F** Serum levels of E_2_, FSH, LH, and AMH (*n* = 10, except for POI group [*n* = 8], BMSCs [*n* = 9]). **G**–**H** Ovarian weight and index (*n* = 10, except for POI group [*n* = 8], BMSCs [*n* = 9]). **I**–**L** The numbers of follicles in different levels (*n* = 6). **M** The numbers of total follicles (*n* = 6). **N** The numbers of atresia follicles (*n* = 6). **O** The structure of ovaries. ★ represents primordial follicles, ▲ represents secondary follicles. Bar: 100, 50 μm.* *P* < 0.05, ** *P* < 0.01, *** *P* < 0.001; **** *P* < 0.0001

We investigated the combinational effects of BMSCs and moxibustion in ovarian tissue restoration (Fig. 5). First, rat estrous cycles of each group were observed. MOX, BMSCs, and BMSCs-MOX reduced the incidence of estrous cycle disorders in rats (Fig. 5B), with BMSCs-MOX showing the most significant effect. Subsequently, the hormone levels in rat serum samples were measured. Compared with the POI group, the MOX group showed a significant increase in serum E_2_ and AMH levels and a significant decrease in serum FSH levels (Fig. 5C–F). BMSCs treatment decreased only the serum FSH levels; in contrast, BMSCs-MOX treatment significantly increased serum E_2_ and AMH levels and reduced FSH and LH levels (Fig. 5C–F). Next, we examined BMSCs-MOX’s impact on ovarian tissue restoration in rats with POI. Compared with those in the POI group, the ovarian weights of rats in the MOX and BMSCs-MOX groups were significantly increased (Fig. 5G); the ovarian indexes were significantly higher in all three treated groups (Fig. 5H). Histopathological analysis revealed that POI rats had significantly reduced numbers of follicles at all levels (Fig. 5I–O). Treatment with MOX, BMSCs, or BMSCs-MOX significantly increased the number of ovarian primordial follicles (Fig. 5I) and reduced the number of atresia follicles (Fig. 5N). The MOX and BMSCs-MOX group showed a significant increase in the number of primary follicles (Fig. 5J), and the BMSCs and BMSCs-MOX groups showed an increase number of secondary follicles (Fig. 5K). Therefore, these observations supported that BMSCs-MOX treatment had superior therapeutic potential for POI compared to MOX or BMSCs treatment.

### BMSCs-MOX mitigates mitochondrial damage and suppresses mitophagy

**Fig. 6:**
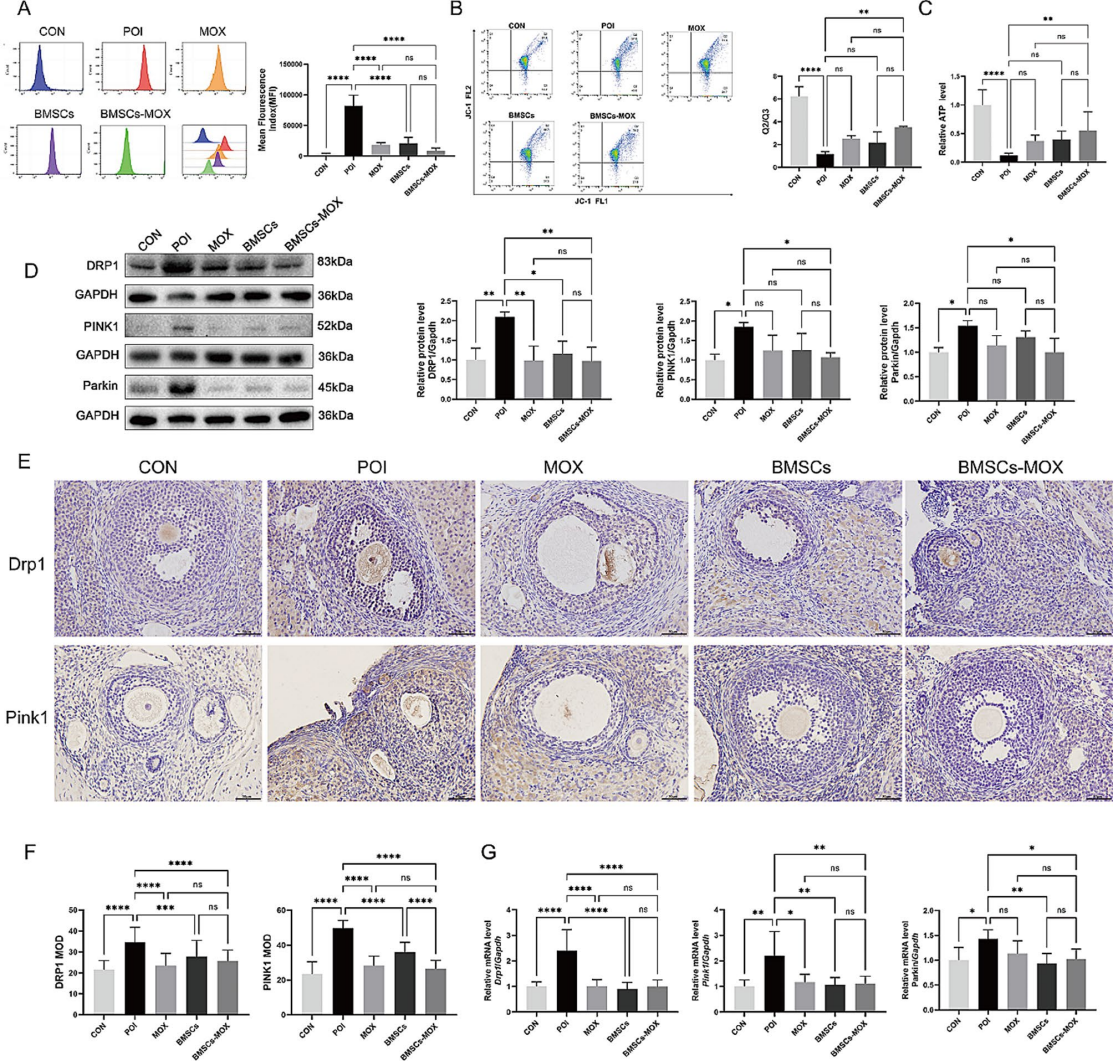
BMSCs-MOX mitigates mitochondrial damage and suppress mitophagy. **A** ROS production. Histograms show the mean fluorescence index (MFI) of DCF-DA levels in each group (*n* = 6). **B** Representative pictures of JC-1 and red/green ratio (Q2/Q3) (*n* = 3). **C** ATP concentration (*n* = 6). **D** DRP1, PINK1, and Parkin protein levels (*n* = 3). Full-length blots are presented in Additional file 4: Fig. S2. **E** Representative IHC for DRP1 and PINK1 (*n* = 6), Bar: 50 μm. **F** MODs of DRP1 and PINK1. G *Drp1*, *Pink1*, and Parkin mRNA expression (*n* = 6). * *p* < 0.05, ** *p* < 0.01, *** *p* < 0.001; **** *p* < 0.0001

To investigate the mechanisms by which BMSCs-MOX restored ovarian aging, we measured intracellular ROS release and mitochondrial function (MMP, ATP) in ovarian GCs. As shown in Fig. 6A, Cy increased intracellular ROS release in GCs, and all three treated groups showed an inhibition of ROS release, with BMSCs-MOX treatment being particularly effective. The excessive accumulation of ROS can disrupt the permeability of the mitochondrial membrane, resulting in a decline in MMP and subsequent mitochondrial dysfunction. JC-1, a widely used fluorescent probe, is employed to detect cellular MMP. When MMP levels are high, JC-1 aggregates in the mitochondria matrix, resulting in red fluorescence (located in Q2). Conversely, when MMP levels are low, JC-1 remains as a monomer and produces green fluorescence (located in Q3). The ratio of red to green fluorescence (Q2/Q3) is indicative of the MMP. Compared with those in the CON group, GCs in the POI group exhibited decreased MMP levels (Fig. 6B). Compared with the POI group, MMP levels showed a higher trend with no significant difference in the MOX and BMSCs groups. In contrast, BMSCs-MOX groups showed a significant elevation in cellular MMP (Fig. 6B). ATP production is a crucial function of mitochondria. In this study, we evaluated mitochondrial dysfunction by measuring ATP levels in GCs. As shown in Fig. 6C, ATP levels were significantly lower in the POI group than in the CON group. No significant difference in the increase in ATP levels in GCs was observed between the MOX and BMSCs groups and the POI group; in contrast, ATP levels in GCs were significantly increased in the BMSCs-MOX group. Furthermore, when compared with the BMSCs-MOX group, ATP levels showed a lower trend with no significant difference in the MOX and BMSCs groups. These observations indicated that BMSCs-MOX mitigated intracellular ROS release and mitochondrial damage.

Moderate mitophagy is involved in eliminating dysfunctional or surplus mitochondria to maintain homeostasis. In this study, we examined the mRNA and protein levels of three well-known genes associated with mitophagy, namely dynamin-related protein 1 (*Drp1*), phosphate and tensin homolog-induced kinase 1 (*Pink1*), and Parkin. Elevated mRNA and protein levels of these genes were observed in the POI group. The MOX, BMSCs, and BMSCs-MOX groups showed significantly decreased mRNA and protein levels of *Drp1* in ovarian tissues (Fig. 6D–G). IHC and qRT-PCR assays demonstrated that MOX and BMSCs decreased protein and mRNA levels of *Pink1* and Parkin in the ovaries (Fig. 6E–G). However, there was only a decreased trend with no statistically significant difference in the Western blot results for Pink1 and Parkin protein levels (Fig. 6D). Interestingly, in the ovaries of the BMSCs-MOX group, the *Pink1* and Parkin mRNA and protein levels were significantly decreased. These findings suggested that BMSCs-MOX inhibited the Pink1/Parkin-dependent mitophagy signaling pathway.

### BMSCs-MOX improves ovarian function by inhibiting the overactivated Pink1/Parkin-dependent mitophagy signaling pathway

**Fig. 7:**
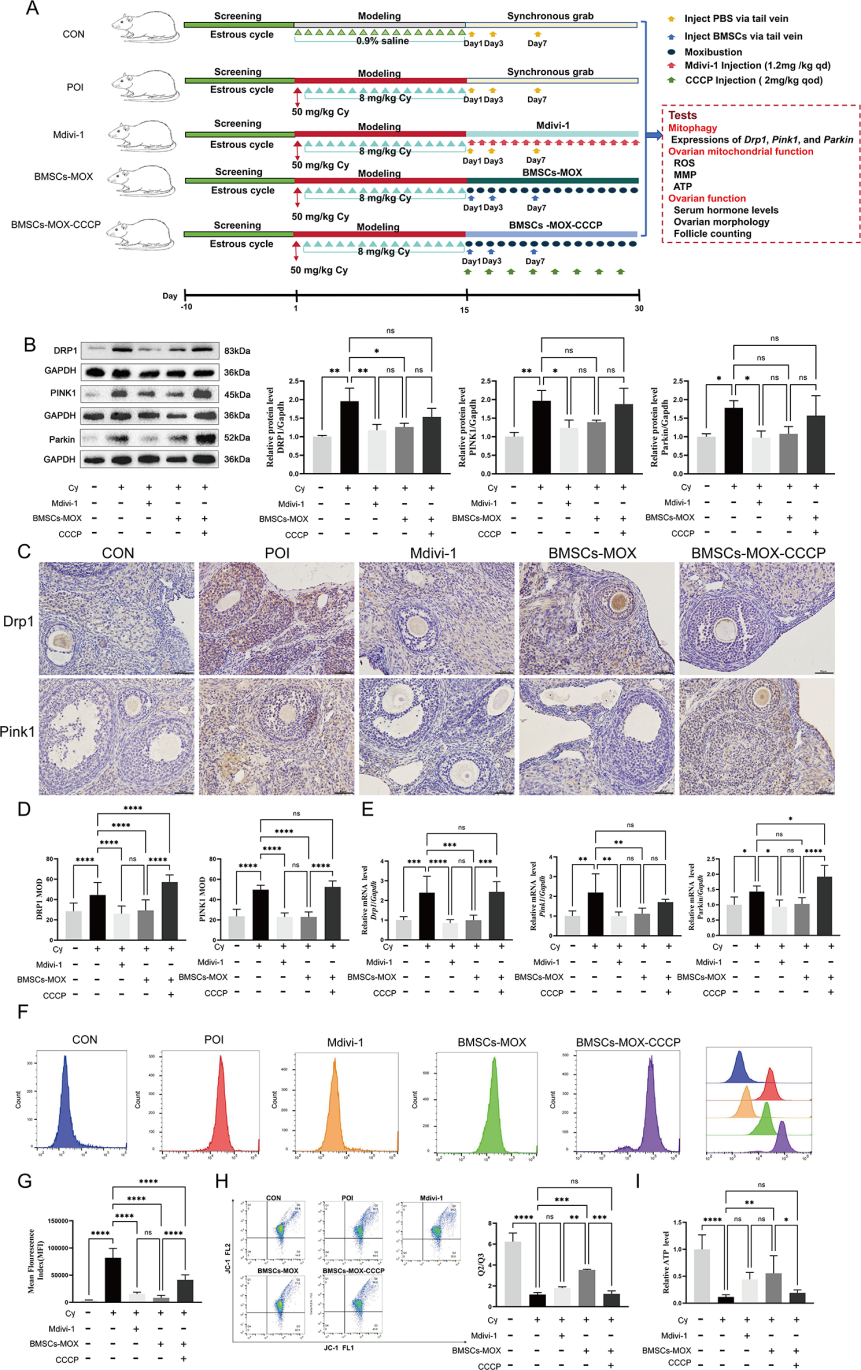
BMSCs-MOX attenuates mitochondrial damage by inhibiting the overactivated Pink1/Parkin-dependent mitophagy signaling pathway. **A** The process of treatment in each group. **B** DRP1, PINK1, and Parkin protein levels (*n* = 3). Full-length blots are presented in Additional file 4: Fig. S2. **C** Representative IHC for DRP1 and PINK1 (*n* = 6), Bar: 50 μm. **D** MODs of DRP1 and PINK1. **E***Drp1*, *Pink1*, and Parkin mRNA expression (*n* = 6). **F**–**G** ROS release (*n* = 6). **H** Representative images of JC-1 and red/green ratio (Q2/Q3)(*n* = 3). **I** ATP concentration (*n* = 6). * *p* < 0.05, ** *p* < 0.01, *** *p* < 0.001; **** *p* < 0.0001

**Fig. 8:**
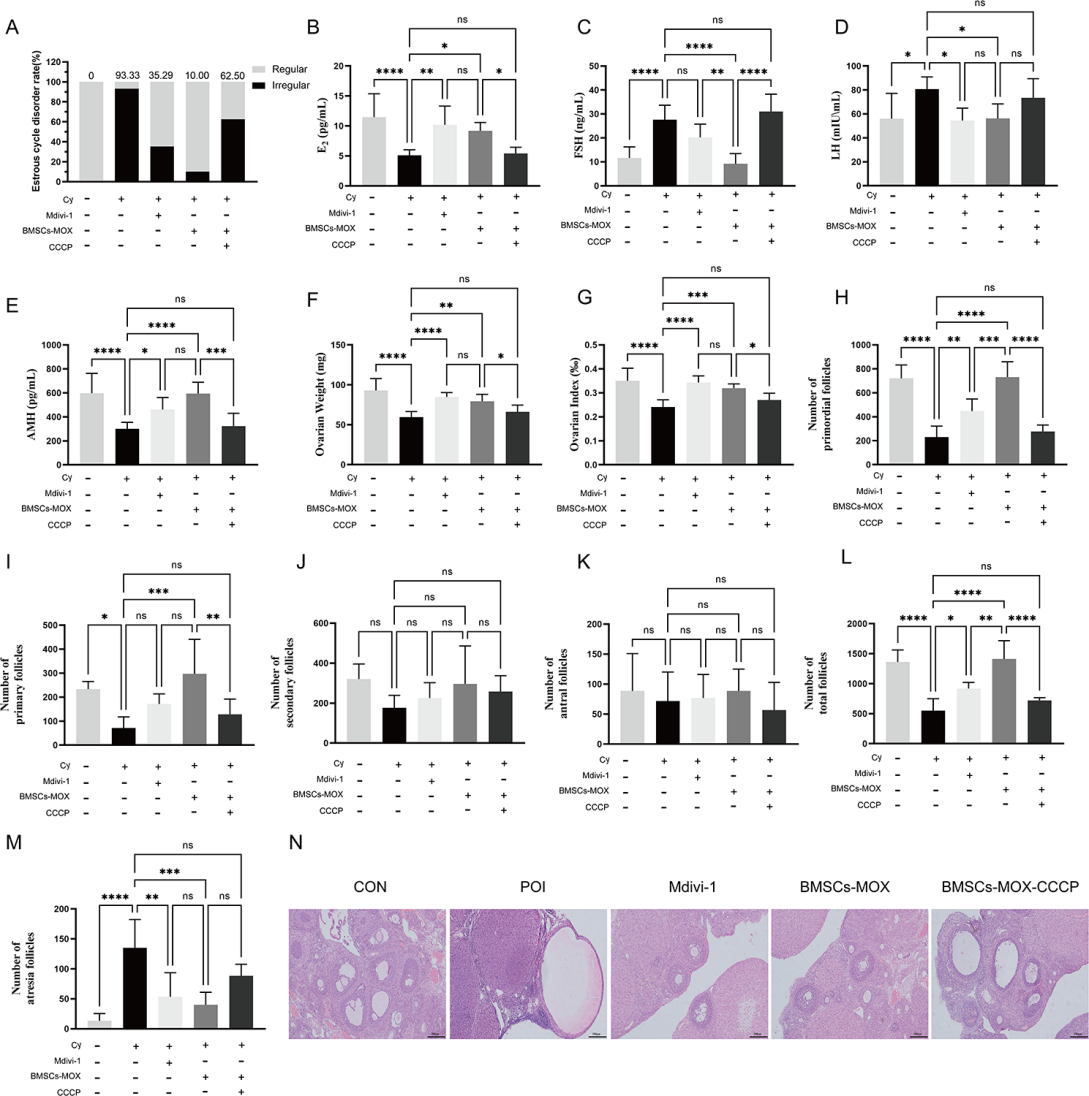
BMSCs-MOX improves ovarian function by inhibiting the overactivated Pink1/Parkin-dependent mitophagy signaling pathway. **A** The rate of estrous cycle disorder (CON group [*n* = 20], POI group [*n* = 15], Mdivi-1 group [*n* = 17], BMSCs-MOX group [*n* = 20], BMSCs-MOX-CCCP group [*n* = 16]). **B**–**E** Serum levels of E_2_, FSH, LH, AMH (*n* = 10). **F**–**G** Ovarian weights and indexes (*n* = 10). **H**–**K** The numbers of follicles in different levels (*n* = 6). **L** The numbers of total follicles (*n* = 6). **M** The numbers of atresia follicles (*n* = 6). **N** The structure of ovaries. Bar: 100 μm. * *p* < 0.05, ** *p* < 0.01, *** *p* < 0.001; **** *p* < 0.0001

To confirm the crucial role of mitophagy in improving ovarian function caused by BMSCs-MOX, both a mitophagy inhibitor (Mdivi-1) and activator (CCCP) were added to test the effects of BMSCs-MOX (Fig. 7). Mdivi-1 significantly reduced *Drp1*, *Pink1*, and Parkin mRNA and protein levels in the ovarian tissues of rats (Fig. 7B–E), indicating that Mdivi-1 effectively inhibited mitophagy. Additionally, Mdivi-1 significantly decreased ROS release and increased the MMP and ATP levels (Fig. 7F–I). As a result, Mdivi-1 corrected endocrine disruption in rats with POI, leading to increased serum E_2_ and AMH levels and decreased serum LH levels (Fig. 8). It also reduced the rate of estrous cycle disorders and increased the number of primordial and total follicles (Fig. 8). Therefore, mitophagy inhibition ameliorated Cy-induced ovarian damage.

When CCCP was added to BMSCs-MOX, a decrease in the modulatory effect of BMSCs-MOX on mitophagy was observed. In the BMSCs-MOX-CCCP group, there were no significant changes in the protein levels of DRP1, PINK1, and Parkin compared to the POI group (Fig. 7B). IHC and qRT-PCR assay indicated that compared to the BMSCs-MOX group, CCCP increased the protein and mRNA levels of *Drp1* and *Pink1* (Fig. 7C–E). Moreover, rats in the BMSCs-MOX-CCCP group exhibited increased ROS levels and decreased ATP and MMP levels in ovarian tissues (Fig. 7F–I). CCCP inhibited the improvement in ovarian function by BMSCs-MOX. Rats in the BMSCs-MOX-CCCP group had a significantly higher rate of estrous cycle disorders than those in the BMSCs-MOX group, along with lower E_2_ and AMH levels and a higher FSH level (Fig. 8A–E). The ovarian morphology analysis revealed a significant reduction in the number of primordial, primary, and total follicles in the BMSCs-MOX-CCCP group compared with that in the BMSCs-MOX group (Fig. 8F–N). These findings supported the fact that BMSCs-MOX enhanced PINK1/Parkin-mediated mitophagy to mitigate ROS release and mitochondrial damage and improve ovarian function.

## Discussion

This work established a POI rat model with a suitable dose of Cy and confirmed that moxibustion treatment upregulated tissue expression of *Sdf1* and *Cxcr4*, increased migration ability of MSCs, and improved ovarian performance after treatment with BMSCs. Further, we investigated the potential mechanisms of BMSCs combined with moxibustion in the treatment of Cy-induced POI. The combination of BMSCs with moxibustion improved ovarian function by alleviating mitochondrial damage and inhibiting excessive mitophagy.

Cy is commonly used to treat various cancers in adolescent women [31], which often leads to gonadal toxicity (such as premature ovarian aging). Previous studies have shown that Cy exposure reduces primordial follicles in rat and mouse ovaries [32, 33]; however, unified and recognized standards for establishing a POI model using Cy are currently unavailable, thereby hindering further research. Considering that those drug-induced ovarian injuries are usually dose-dependent, we established a rat model of chronic Cy-induced POI using three different doses of Cy. Given the survival rate and stabilization effect, a medium-dose Cy (D1: 50 mg/kg, D2–D15: 8 mg/kg maintenance) injection is more suitable to establish a Cy-induced POI rat model. Rats exposed to medium-dose Cy exhibited conspicuous and chronic ovarian damage, including estrous cycle disorders, disrupted serum hormone levels, reduction in primordial follicles, and increase in atretic follicles, consistent with the clinical manifestations of POI.

Definitely, MSCs have emerged as a valuable tool in effectively improving ovarian function [34–36]. In 2018, Gupta et al. successfully aided a 45-year-old woman conceive and deliver a healthy baby girl using autologous BMSCs transplantation therapy, which is believed to be the world’s first successful application of BMSCs in a perimenopausal woman to deliver a healthy baby [37]. Herraiz et al. conducted a study involving 17 patients (< 39 years of age) with decreased ovarian reserve who received BMSCs transplantation [38]. They observed improved ovarian function and increased sinus follicle counts in approximately 80% of the patients within 4 weeks. At the time of their article’s publication, five patients had become pregnant, and three of them gave birth to healthy babies. It is evident that MSCs therapy has demonstrated noteworthy advancements in clinical applications. However, it is crucial to acknowledge that the desired outcomes in ovarian rejuvenation with MSCs have not been fully achieved, and thus it remains a major area of debate in this field. The primary reason for this discrepancy is widely attributed to the inefficient homing and inadequate survival of MSCs following transplantation [39]. Recruiting more MSCs and enhancing their survival in damaged ovaries can effectively improve their therapeutic effect. The motility of MSCs is primarily regulated by *Sdf1* and its receptor *Cxcr4* [40, 41]. *Sdf1* is highly expressed in damaged tissues, while *Cxcr4* is highly expressed in BMSCs [42]. Enhancing *Sdf1* and *Cxcr4* expression has been shown to promote the mobilization and homing of MSCs. While viral transduction, drug pretreatment, and gene editing are important methods to explore in promoting expression of *Sdf1* and *Cxcr4*, they may not currently be feasible in clinical practice [12, 43, 44]. Moxibustion, an external treatment used in traditional Chinese medicine, is popular in clinical practice and is easy to use. Several studies have indicated that moxibustion can alleviate fatigue in the patient’s body [45] and reduce oxidative stress [19] and inflammation [28] in ovarian tissues to enhance ovarian reserve. Notably in this study, the addition of moxibustion significantly increased and prolonged the duration of BMSCs in the tissues of the BMSC-MOX group, suggesting that moxibustion intervention improved the homing and survival efficiency of BMSCs. The higher levels of *Sdf1* and *Cxcr4* in the ovarian tissues further supported these findings. In addition, subsequent tests for ovarian function also showed that the combination therapy of BMSCs and moxibustion was significantly superior to BMSCs transplantation alone in reducing ovarian injury. The current findings have clear translational implications for improving the efficacy of stem cell therapy in patients with POI.

Mitochondrial dysfunction has been reported to be responsible for the progression of ovarian aging [46]. It alters the function of ovarian GCs and premature aging of oocytes. Exposure to Cy quickly elevates levels of ROS within the ovary, thus reducing MMP and mitochondrial energy production [23, 28]. Mitophagy, an essential mechanism for removing damaged mitochondria and maintaining cellular homeostasis, plays a central role in regulating mitochondrial quality [47]. Mitophagy has both beneficial and harmful effects. At a basal level, mitophagy plays a key role in removing damaged mitochondria to maintain cellular and mitochondrial homeostasis, which is advantageous to cell survival and function [48]. In age-related ovarian diseases, mitophagy is often reduced, and enhancing mitophagy can help improve disease conditions by eliminating damaged mitochondria and promoting cellular homeostasis [49]. In these diseases, chronic stress, which results to ovarian aging, tend to be less toxic but persistent. In contrast, our study suggests that Cy promotes mitophagy-mediated excessive ovarian toxicity. We hypothesize that the high sensitivity, strong toxicity, and prolonged exposure of ovarian cells to Cy contribute to excessive and deleterious mitogaphy. This excessive mitogaphy not only eliminates damaged mitochondria but also results in the removal of healthy mitochondria, ultimately causing abnormal cellular function or cell death, which in turn contributes to ovarian aging. Importantly, our research outlines the classical Pink1/Parkin pathway of mitophagy through which Cy regulates excessive mitophagy in ovarian toxicity. Cy exposure activated DRP1 relocation from the cytoplasm to the division site near the outer mitochondrial membrane, initiating mitochondrial fission [50]. Mitochondrial fragmentation and altered membrane potential lead to PINK1 accumulation in the outer mitochondrial membrane, which recruits and activates Parkin, leading to the ubiquitination of mitochondrial surface proteins and triggering mitophagy [51]. In our study, the mitophagy inhibitor Mdivi-1 [52] effectively suppressed the overactivation of mitophagy and improved ovarian function in rats. Therefore, we hypothesize that exposure to Cy induces abnormal mitochondrial fission and dysfunction, which activate mitophagy. However, prolonged and high-intensity use of Cy may cause sustained over-activation of mitophagy, ultimately impairing ovarian function. Controlling mitophagy levels can ameliorate Cy-induced ovarian damage. The study shows that BMSCs-MOX can enhance ovarian function and reduce ovarian mitochondrial damage, possibly through the regulation of Pink1/parkin-dependent mitophagy. This hypothesis was further supported by the notable decrease in the effects of BMSCs-MOX after treatment with the mitophagy agonist CCCP.

However, there are certain limitations in our study. First, the study was conducted using a rat model, which is not as closely related to the human body as the primate model. Although rat and human ovaries have significant structural and functional similarities, it is important to conduct further studies using ovarian organoids or primate models to validate these findings in the future. Additionally, our investigation of mitophagy was limited to the classical pathway. In future research, we plan to explore the non-classical pathway and assess the impact of autophagic flux on the ovarian function to gain a better understanding of the role of mitophagy in ovarian aging.

## Conclusions

Our research indicates that moxibustion enhances the migration, homing, and survival of transplanted BMSCs, as well as their ability to repair ovarian damage. Furthermore, our findings shed light on the mechanisms of Cy-induced ovarian damage, that is, impairing mitochondrial function and activating sustained PINK1/Parkin-dependent mitophagy. The combination of BMSCs and moxibustion therapy effectively reduced the excessive activation of mitophagy, which helped prevent mitochondrial damage, ultimately leading to an improvement in ovarian function. These results may help identify novel therapeutic targets and medications for POI, as well as offer insights into enhancing the efficacy of stem cell therapy for patients with POI.

### Electronic supplementary material

Below is the link to the electronic supplementary material.

Supplementary Material 1

Supplementary Material 2

Supplementary Material 3

Supplementary Material 4

## Data Availability

All data reported in this paper are available from the corresponding author upon request.

## Abbreviations

AMH: Anti-Müllerian hormone
BMSCs: Bone marrow mesenchymal stem cells
Cxcr4: C-X-C chemokine receptor type 4
Cy: Cyclophosphamide
Drp1: Dynamin-related protein 1
E_2_: estradiol
EA: Electroacupuncture
FSH: Follicle-stimulating hormone
GCs: Granulosa cells
H&E: Hematoxylin & eosin
IHC: Immunohistochemistry
LH: Luteinizing hormone
MMP: Mitochondrial membrane potential
MOD: Mean optical density
MSCs: Mesenchymal stem cells
PBS: phosphate-buffered saline
PCR: polymerase chain reaction
Pink1: Phosphate and tensin homolog-induced kinase 1
POI: Premature ovarian insufficiency
qRT-PCR: Quantitative reverse-transcription polymerase chain reaction
ROS: Reactive oxygen species
Sdf1: Stromal cell-derived factor 1
