# Supplementary material for: Combination of bone marrow mesenchymal stem cells and moxibustion restores cyclophosphamide-induced premature ovarian insufficiency by improving mitochondrial function and regulating mitophagy
Source: Stem Cell Res Ther. 2024 Apr 8;15:102. doi: 10.1186/s13287-024-03709-0 (PMC11003045; doi:10.1186/s13287-024-03709-0)
Supplement: Supplementary file 1 — Supplementary Material 1 [file 13287_2024_3709_MOESM1_ESM.docx]

**Table S1.** Antibodies

| **Table S1. Antibodies** | | | | |
| --- | --- | --- | --- | --- |
| **Antibody** | **Company** | **Catalog number** | **Source** | **Dilutions** |
| GAPDH | FUDE | FD0063 | Mouse | WB：1：5000 |
| CXCR4 | Proteintech | 11073-2-AP | Rabbit | IHC：1：100  WB：1：1000 |
| SDF1 | Proteintech | WL02283 | Rabbit | IHC：1：100  WB：1：1000 |
| DRP1 | Abcam | ERP19274 | Rabbit | WB：1：1000  IHC：1：200 |
| PINK1 | Proteintech | 23274-1-AP | Rabbit | WB：1：1000  IHC：1：800 |
| Parkin | Santa cruz | sc32282 | Mouse | WB：1：1000 |
