# Supplementary material for: Combination of bone marrow mesenchymal stem cells and moxibustion restores cyclophosphamide-induced premature ovarian insufficiency by improving mitochondrial function and regulating mitophagy
Source: Stem Cell Res Ther. 2024 Apr 8;15:102. doi: 10.1186/s13287-024-03709-0 (PMC11003045; doi:10.1186/s13287-024-03709-0)
Supplement: Supplementary file 2 — Supplementary Material 2 [file 13287_2024_3709_MOESM2_ESM.docx]

**Table S2.** Sequence of qRT-PCR primers.

| **Table S2. Sequence of qRT-PCR primers.** | | | |
| --- | --- | --- | --- |
| **Name** |  | **Sequence(5'-3')** | **Length(bp)** |
| *Gapdh* | Forward | GACATGCCGCCTGGAGAAAC | 92 |
|  | Reverse | AGCCCAGGATGCCCTTTAGT |  |
| *Cxcr4* | Forward | CAGCCTGTGGATGGTGGTGTTC | 112 |
|  | Reverse | GGAGTGTGACAGCTTGGAGATGATG |  |
| *Sdf1* | Forward | CGCTCTGCATCAGTGACGGTAAG | 128 |
|  | Reverse | AAGGGCACAGTTTGGAGTGTTGAG |  |
| *Drp1* | Forward | ACAGCGTCCCAAAGGCAGTAATG | 129 |
|  | Reverse | CCATGTCCTCGGATTCAGTCAGAAG |  |
| *Pink1* | Forward | GTATGAAGCCACCATGCCCACAC | 101 |
|  | Reverse | CATCTGCTCCCTTTGAGACGACATC |  |
| Parkin | Forward | CGCCACGTGATCTGTTTGGA | 80 |
|  | Reverse | TGAGCGTCGTGGACAAACTG |  |
