# Supplementary material for: Combination of bone marrow mesenchymal stem cells and moxibustion restores cyclophosphamide-induced premature ovarian insufficiency by improving mitochondrial function and regulating mitophagy
Source: Stem Cell Res Ther. 2024 Apr 8;15:102. doi: 10.1186/s13287-024-03709-0 (PMC11003045; doi:10.1186/s13287-024-03709-0)
Supplement: Supplementary file 3 — Supplementary Material 3 [file 13287_2024_3709_MOESM3_ESM.docx]

**
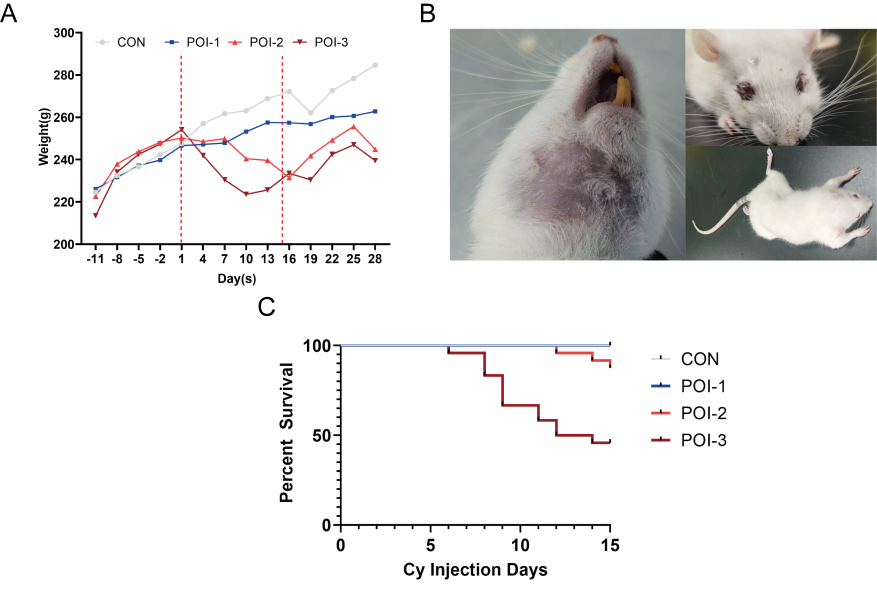
**

**Fig. S1. Cyclophosphamide leads to acute systemic injury in rats. A** Effect of Cy on body weight. **B** Representative picture of rats with depression, fur shedding, and mucous membrane hemorrhage. **C** Survival rates.
