# Supplementary material for: Combination of bone marrow mesenchymal stem cells and moxibustion restores cyclophosphamide-induced premature ovarian insufficiency by improving mitochondrial function and regulating mitophagy
Source: Stem Cell Res Ther. 2024 Apr 8;15:102. doi: 10.1186/s13287-024-03709-0 (PMC11003045; doi:10.1186/s13287-024-03709-0)

**Fig. S2.Full-length blots.**

**Fig. 4C Full-length blots**

1 POI

2 BMSCs

3 BMSCs-MOX


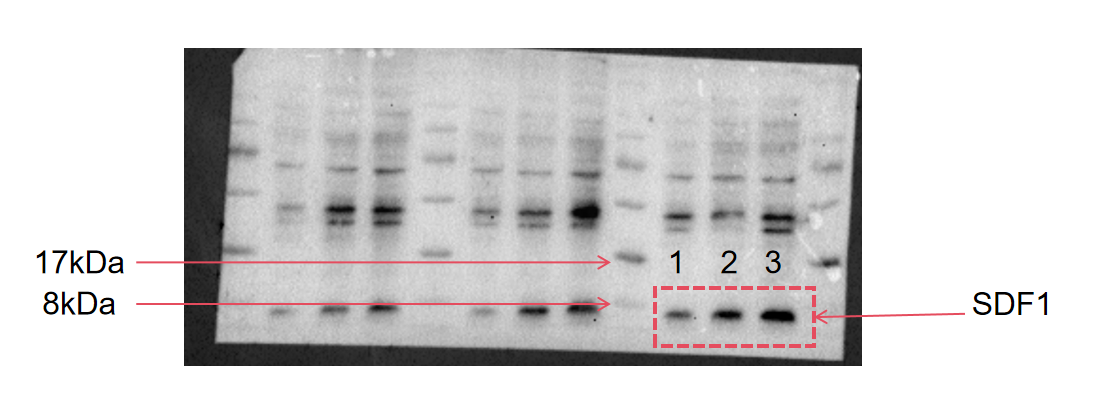


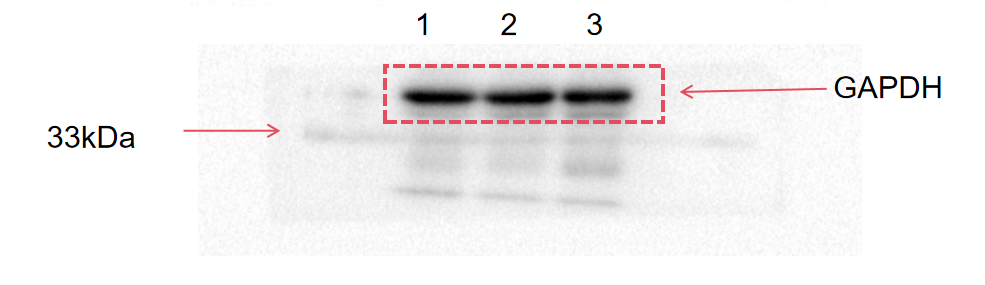


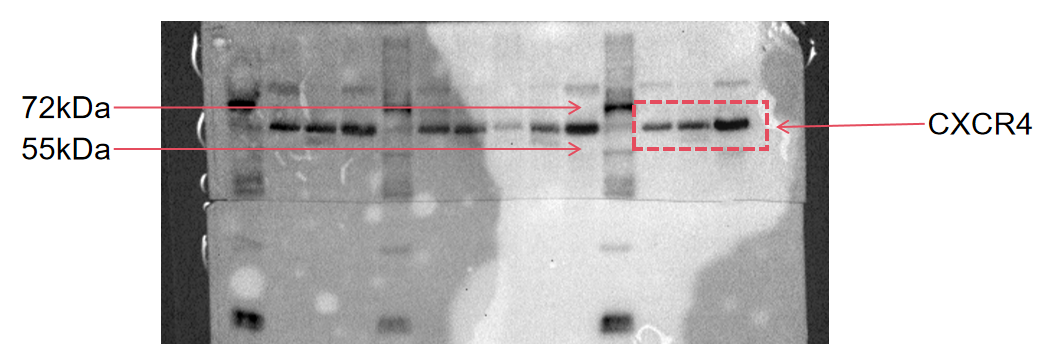


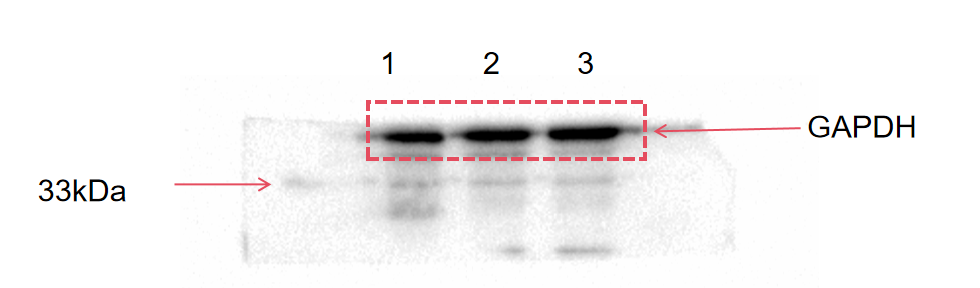


**Fig. 6D Full-length blots**

1 CON

2 POI

3 MOX

4 BMSCs

5 BMSCs-MOX


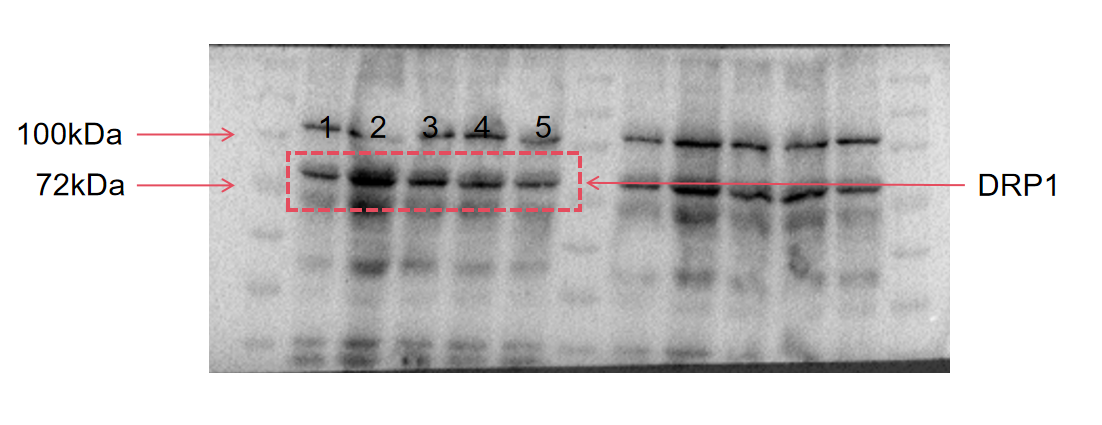


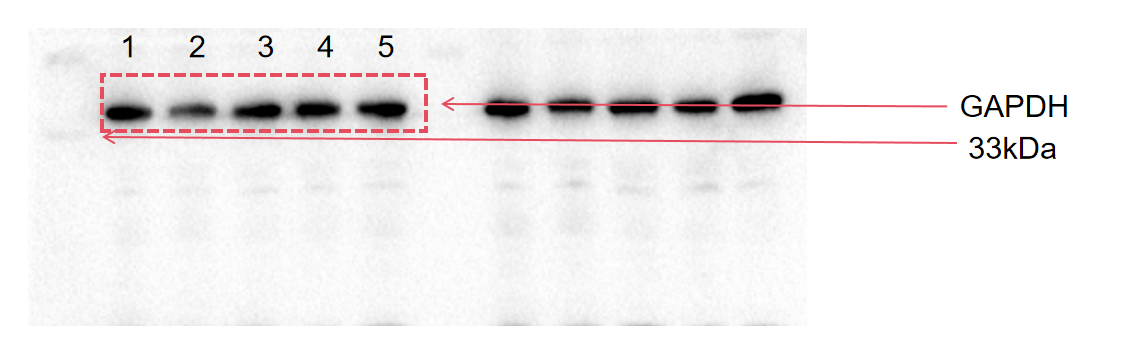


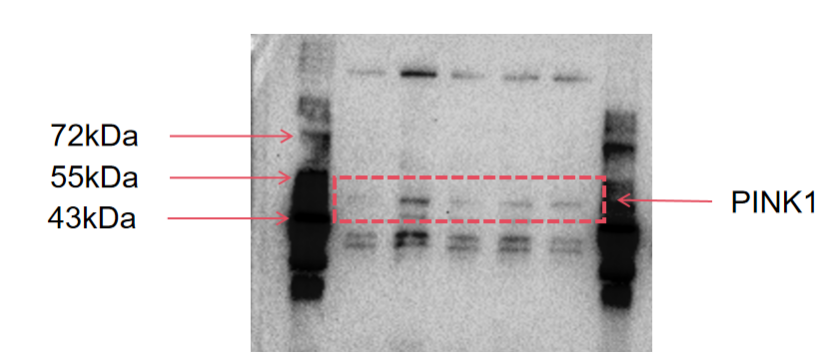


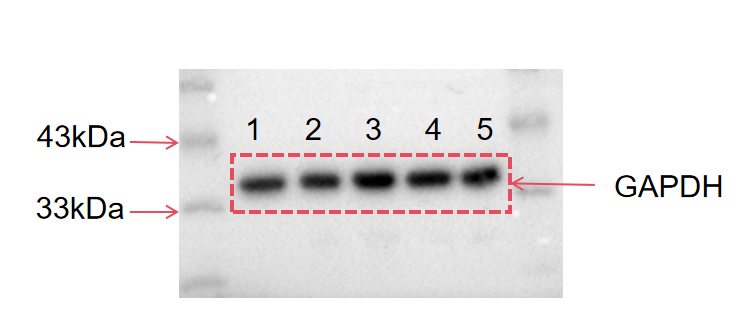


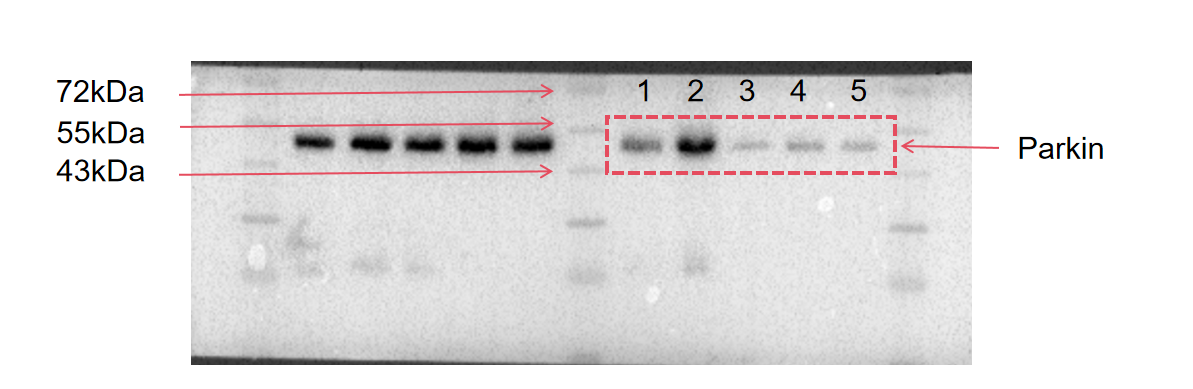


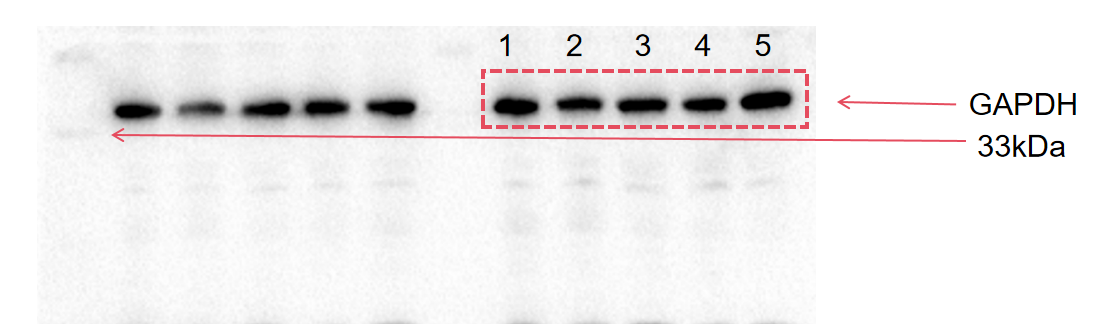


**Fig. 7D Full-length blots**

1 CON

2 POI

3 Mdivi-1

4 BMSCs-MOX

5 BMSCs-MOX-CCCP


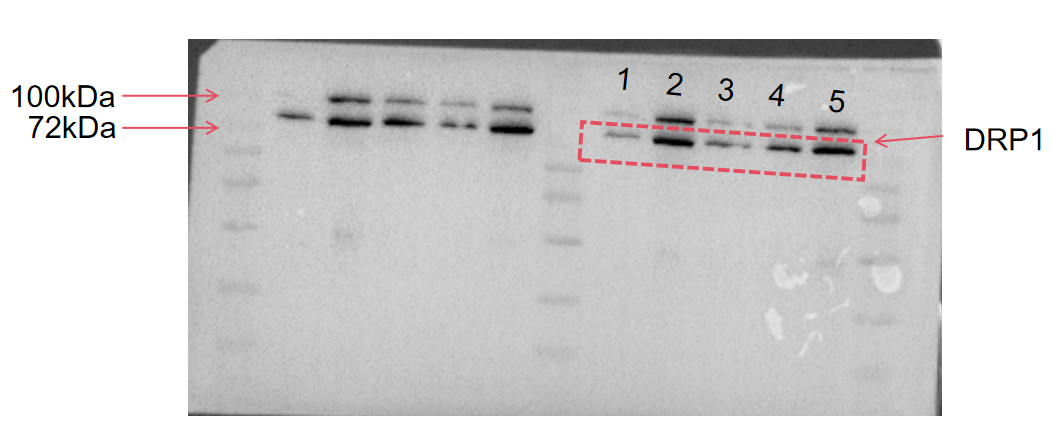


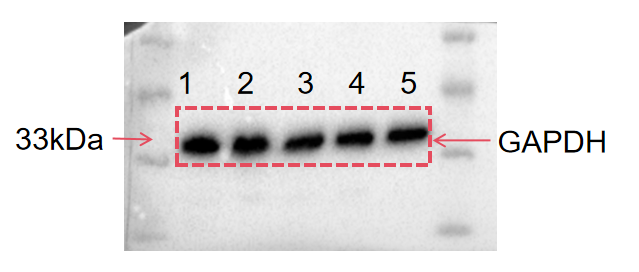


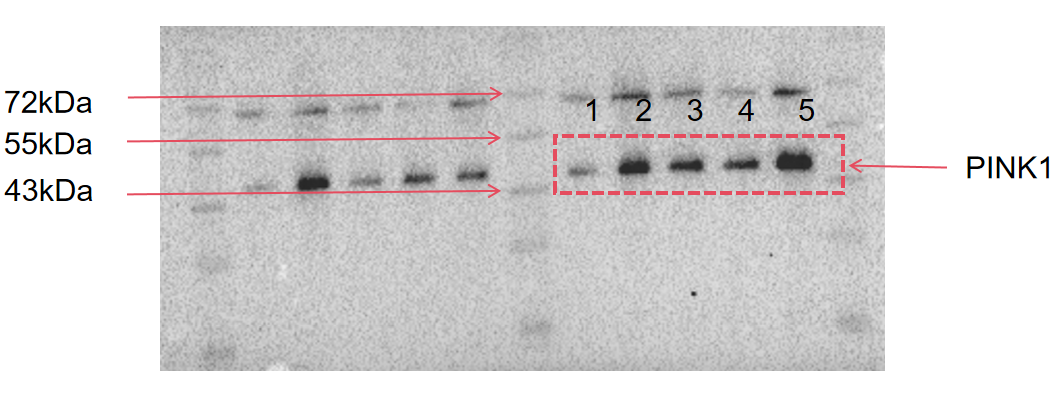

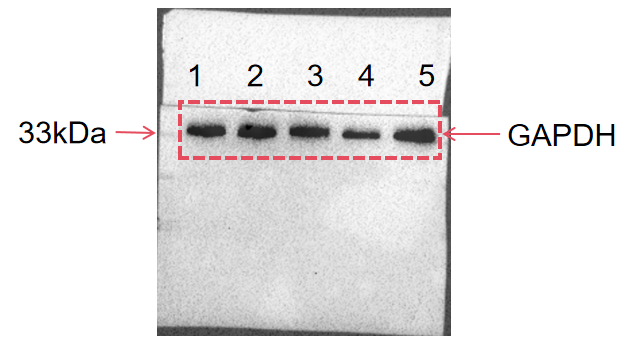


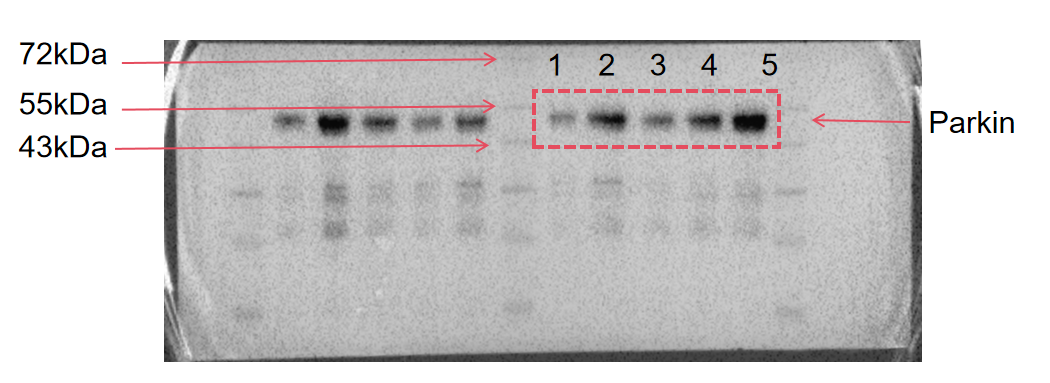


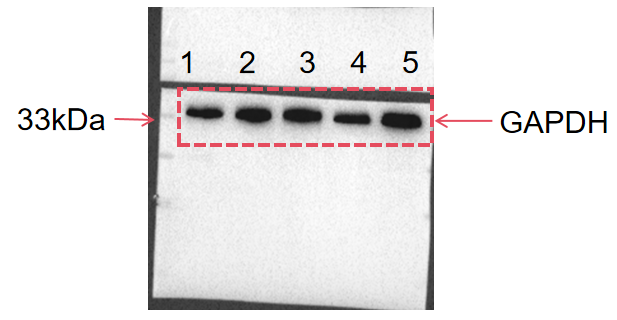

Supplement: Supplementary file 4 — Supplementary Material 4 [file 13287_2024_3709_MOESM4_ESM.docx]
